# Looking at randomized trials with the critical eyes of epidemiologists: the case of screening colonoscopy

**DOI:** 10.1007/s10654-025-01269-y

**Published:** 2025-07-08

**Authors:** Hermann Brenner, Thomas Heisser, Michael Hoffmeister

**Affiliations:** 1 Division of Clinical Epidemiology and Aging Research, German Cancer Research Center (DKFZ), INF 581, 69120 Heidelberg, Germany; 2 German Cancer Consortium (DKTK), German Cancer Research Center (DKFZ), Heidelberg, Germany; 3 Medical Faculty Heidelberg, Heidelberg University, Heidelberg, Germany

## Abstract

Based on compelling evidence from observational epidemiological studies, screening colonoscopy has since long been thought to strongly lower the burden of colorectal cancer (CRC), both by early detection of prevalent CRC and prevention of incident CRC through detection and removal of precancerous lesions. Widespread offer and use of screening colonoscopy went along with a dramatic decline in CRC incidence in screening age groups in the US, in contrast to an increase in incidence at younger ages and in countries not engaging in CRC screening. The recently published 10-year results from the NordICC trial, the first randomized clinical trial (RCT) reporting long-term effects of screening colonoscopy on CRC risk and mortality, has been widely interpreted as challenging the evidence for strong efficacy of screening colonoscopy. Such reasoning was based on the trust that randomization in this large-sized trial should have prevented any residual confounding that might have affected the observational epidemiological studies. However, randomization cannot prevent other potential biases which should be carefully addressed and minimized in both observational and interventional studies. We illustrate that such biases may have led to major underestimation of screening effects in the NordICC trial. The observed patterns underline the need for more rigorous efforts to prevent and correct for such biases, along with the need to derive more informative metrics of screening efficacy. Such metrics should include informative estimates of screening colonoscopy effects on both early detection of prevalent CRC cases and prevention of incident CRC cases. The momentum for CRC screening should by no means slowed by misinterpretation of the NordICC trial evidence.

## Background

Randomized controlled trials (RCTs) are commonly considered as providing the necessary high-level evidence to confirm effectiveness of medical interventions. Their main and unique asset compared to observational epidemiological studies is that, provided sufficiently large sample size, the randomization should prevent confounding by unmeasured or imperfectly measured confounding factors. Even though high quality observational epidemiological studies make major efforts to prevent relevant confounding by sophisticated approaches in study design, data collection and data analysis, residual confounding by unmeasured or imperfectly measured confounders can never be completely ruled out.

However, confounding is just one of multiple potential sources of bias in studies on the effects of medical interventions. Excluding or minimizing other sources of bias, such as selection bias or exposure and outcome information bias may be equally relevant or even more relevant for deriving valid effect estimates. Several of these sources of bias may not only affect the validity of observational epidemiological studies, but may also compromize the validity of RCT based effect estimates. However, while there has been a long tradition in thorough assessment, prevention, correction and discussion of various biases in observational epidemiological studies, possible biases have received much less attention in the realm of RCTs, possibly by taking validity for granted given prevention of confounding by the randomized design. This also seems to have been the case for the recently reported results of the Nordic-European Initiative on Colorectal Cancer (NordICC) trial, the first and so far only RCT reporting effects of screening colonoscopy on incidence and mortality from colorectal cancer (CRC). First results of both intention-to-screen analyses and per-protocol analyses were published in October 2022 [1], and alternative per-protocol analyses have been presented a recent issue of the journal [2]. Although the trial reported a significant reduction of CRC incidence, the effect was much weaker than expected, and no significant reduction of CRC mortality was observed in intention-to-screen analysis. In the following, we line out how potential biases may have led to major underestimation of the effects of screening colonoscopy in this trial, and why more rigorous efforts are needed to prevent and overcome possible major biases in the analysis and interpretation of the trial results.

### Epidemiology of and screening for colorectal cancer

CRC is the third most common cancer and the second most common cause of cancer-related death globally [3]. Prognosis strongly depends on stage at diagnosis, with 5-year relative survival ranging from more than 90% for patients diagnosed with local stage CRC to less than 20% for those diagnosed at distant stage [4], supporting a major role of early detection in lowering the burden of the disease. Furthermore, most CRCs develop slowly over many years through the adenoma-carcinoma sequence, which offers unique opportunities of prevention by endoscopic detection and removal of precancerous lesions.

Following a landmark publication from the US National Polyp Study in 1993 [5], which demonstrated 76% and 88% lower incidence of CRC among participants with colonoscopic polypectomy compared to the average-risk population and people with unresected polyps in an observational longitudinal cohort study design (Table 1), uptake of screening colonoscopy rapidly increased in the US. Meanwhile, more than 65% of the US population above age 55 have had a screening colonoscopy within the past 10 years [4], the most widely recommended screening interval for this screening exam [6]. Since the 1990s, age adjusted CRC incidence almost halved in the US [4]. This decline was exclusively seen in people above 50 years of age, whereas incidence substantially increased in younger, pre-screening ages. Major decreases in CRC incidence above age 50, along with increases below age 50, have also been observed in other countries offering screening colonoscopy, such as Germany, whereas CRC incidence kept rising in countries that did not or did only very recently engage in CRC screening activities [7].

**Table 1:** Examples of large-scale observational epidemiological studies and meta-analyses on the effects of screening colonoscopy reported since 1993

Authors, year (reference)	Study	Relative risk (95% CI)	
		CRC Incidence	CRC mortality
Winawer et al. 1993 [5]	US National Polyp Study	0.24^a^, *p* < 0.001 0.12^b^, *p* < 0.001	
Nishihara et al. 2013 [8]	Nurses' Health Study and Health Professionals Follow-up Study		0.18 (0.10–0.31)
Brenner et al. 2014 [9]	Meta-analysis	0.31 (0.12–0.77)	0.32 (0.23–0.43)
Stock et al. 2016 [10]	Population-wide Canadian cohort		0.36 (0.33–0.38)
Doubeni et al. 2018 [11]	Kaiser Permanente members, US		0.33 (0.21–0.52)
Guo et al. 2021 [12]	ESTHER cohort study, Germany	0.44 (0.33–0.57)	0.34 (0.21–0.53)

^a^Comparison of a cohort with colonoscopic polypectomy with the general population (in which screening colonoscopy was uncommon during the period of investigation); given the inherently increased risk of people with polyps, this comparison underestimates the effects that would have be obtained by comparing an entire screening colonoscopy cohort with the general population

^b^Comparison of a cohort with colonoscopic polypectomy with a retrospective cohort of participants with colonoscopy-detected but unremoved polyps

Since the late 1990 s, multiple observational epidemiological studies, including multiple large-scale cohort studies, have consistently reported strongly reduced CRC incidence and mortality among people who had a screening colonoscopy or flexible sigmoidoscopy (which visualizes the distal colon and rectum where more than 60% of CRCs are located) compared to people who did not use such screening (Table 1 [8–12]). In meta-analyses of such studies published up to 2013 [9], use of screening colonoscopy was associated with an almost 70% reduction of both CRC incidence and CRC mortality, and similarly strong associations have quite consistently been observed in multiple more recent large-scale studies. RCT-based evidence for the effectiveness of sigmoidoscopy screening in reducing CRC incidence and mortality has been consistently established since 2010 by four large-scale RCTs [13–17]. However, only in late 2022, first RCT results on long-term effects of screening colonoscopy became available from the NordICC trial [1].

### Design features and reported results of the NordICC trial

**Fig. 1:**
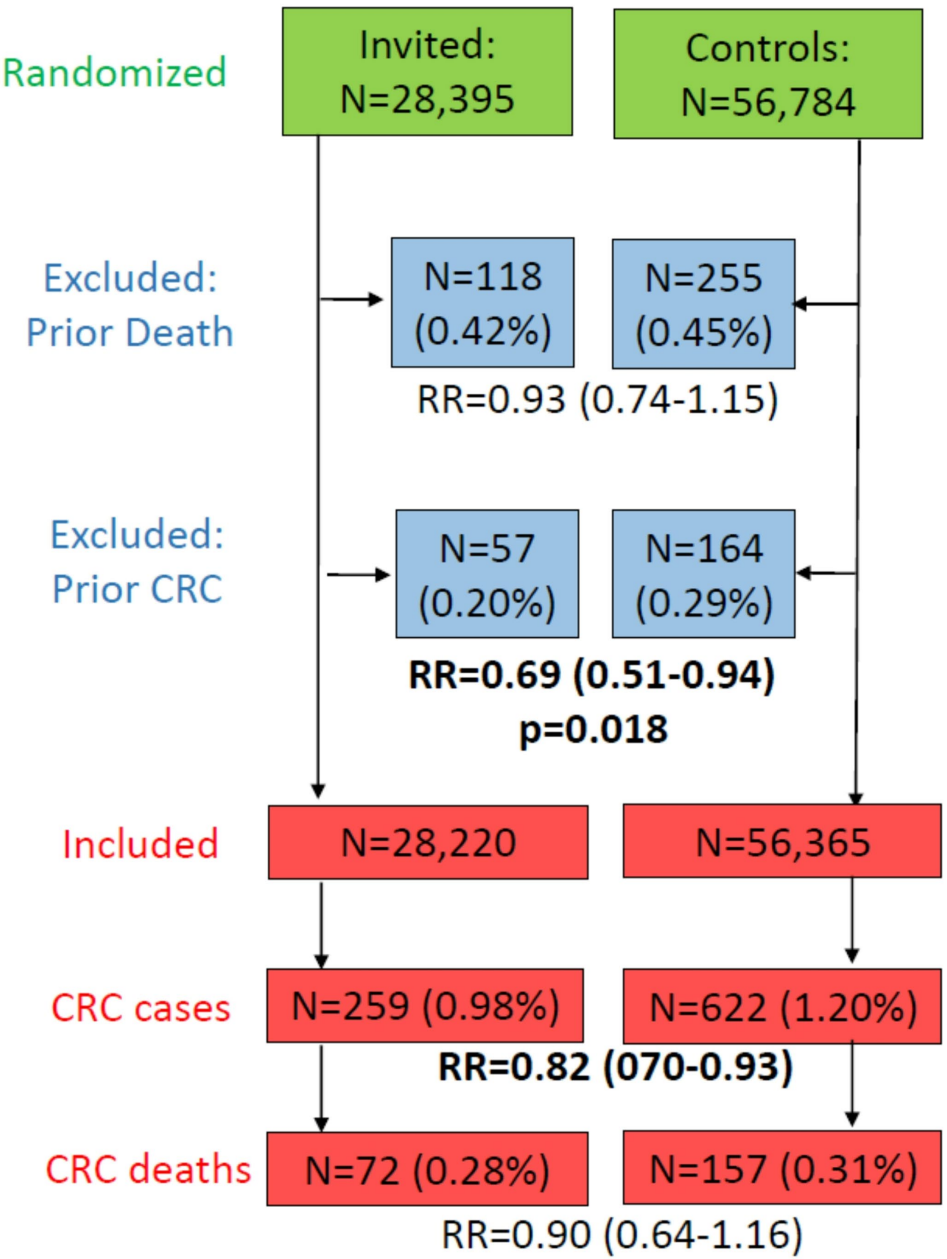
Randomization, post-randomization exclusions and 10-year follow-up results of the NordICC trial

Key design features and results of the NordICC trial are summarized in Table 2; Fig. 1. In this pragmatic RCT, which was intended to estimate the benefit of the offer of screening colonoscopy on the population level, men and women aged 55–64 were directly identified through population registries in four North European countries (Poland, Norway, Sweden and the Netherlands). A total of 94,959 men and women without a known previous CRC diagnosis and without previous screening were randomized in a 1 to 2 ratio to receive an invitation to a single screening colonoscopy or to usual care. Primary endpoints were risk of and death from CRC after a median follow-up of 10 to 15 years. Results after a median 10-year follow up were reported in October 2022 and based on 84,585 participants from Poland (*N* = 54,258), Norway (*N* = 26,411) and Sweden (*N* = 3646). Data from the Netherlands could not be included in the analysis due to confidentiality issues. Among 28,220 participants invited to screening colonoscopy, 42% accepted the offer, but this percentage varied widely between 33% in Poland and 61% in Norway.

**Table 2:** Key design features of the NordICC trial on the effects of the offer of screening colonoscopy on CRC risk and mortality

Design	Pragmatic randomized clinical trial
Intervention	Invitation to a single sceening colonoscopy vs. no invitation
Primary endpoint	CRC risk and CRC mortality after median follow-up of 10–15 years
Secondary endpoints	All-cause mortality after median follow-up of 10–15 years
Study population	Men and women aged 55–64 years with no prior CRC and no prior CRC screening
Countries	Poland, Norway, Sweden, the Netherlands^a^
Recruitment	2009–2014
Randomization	1 (invited, *N* = 31,589) to 2 (usual-care, *N* = 63,370) ratio
Study population included in publication of 10-year results	Total *N* = 84,585, Invited *N* = 28,220, Unsual Care *N* = 56,365; Total N by country: Poland 54,258; Norway 26,411; Sweden 3646
Use of screening offer	42% (ranging from 33% in Poland to 61% in Norway)
Follow-up	Through cancer and population registries
Publication of 10-year results	October 2022
Closing date of follow-up	Not reported

*CRC* colorectal cancer

^a^Data from the Netherlands not included in the 2022 report of 10-year follow-up results due to confidentiality issues

After the 10-year follow-up, 259 participants in the invited group and 622 participants in the usual-care group had a CRC diagnosis, and 72 and 157 had died from CRC, resulting in estimates of relative risk of CRC and of CRC death of 0.82 (95% CI 0.70–0.93) and 0.90 (95% CI 0.64–1.16), respectively, in intention-to-screen analysis (Fig. 1). These effect estimates were much lower than anticipated, and they prompted major doubts and discussion around the use of screening colonoscopy [18].

In addition to the intention-to-screen analyses, per-protocol analyses were performed to estimate the effect of screening if all the participants who were randomly assigned to screening had actually undergone screening. Various methods were used in the original publication in 2022 [1] and a re-analysis published in a recent issue of this journal (2) to adjust the per-protocol estimates for potential risk differences between participants who accepted the screening offer and those who did not. The results of these analyses are summarized in Table 3. Adjusted per-protocol estimates of relative risk (95% CI) varied from 0.59 (0.30–0.98) to 0.69 (0.55–0.83) for CRC risk and from 0.50 (0.27–0.77) to 0.79 (0.24–1.42) for CRC death, with some of the estimates for CRC death having extremely wide confidence intervals, ranging up to 0.00-3.70.

**Table 3:** Results of per-protocol analysis reported in the original NordICC trial report by Bretthauer et al. [1] and the re-analysis by Shi et al. [2]

Publication, year (reference)	Analysis	Relative risk (95% CI)	
		CRC incidence	CRC mortality
Bretthauer et al. 2022 [1]	Intention-to-screen	0.82 (0.70–0.93)	0.90 (0.64–1.16)
	Adjusted per-protocol, main analysis^a^	0.69 (0.55–0.83)	0.50 (0.27–0.77)
	Adjusted per-protocol, sensitivity analysis^b^	0.66 (0.46–0.86)	0.72 (0.00-3.70)
Shi et al. 2025 [2]	Adjusted per-protocol, instrumental variable analysis^c^	0.59 (0.30–0.98) 0.65 (0.48–0.87)	0.71 (0.31–2.89) to 0.79 (0.24–1.42)

^a^Adjusted for baseline covariates: age at randomization, sex, country, duration of follow-up

^b^Adjusted by the method proposed by Cuzick et al. [19]

^c^Instrumental variable analyses with different assumptions: additive homogeneity, multiplicative homogeneity, and monotonicity

### Examples of sources and magnitude of bias

#### Selection bias: differential post-randomization exclusions

Prevention of confounding is a key strength of large-scale randomized trials, and should have led to a balanced distribution of key risk factors also in the NordICC trial. Randomization should also have ensured balanced proportions of post-randomization exclusions of participants not meeting the inclusion criteria, such as participants for whom a previous CRC diagnosis became known after the randomization only. However, as can be seen from Fig. 1 and recently addressed in more detail elsewhere [20], this was not the case. In particular, a significantly lower proportion of people were later excluded from the invited group than from the control group due to a prior CRC (0.20% versus 0.29%, *p* = 0.018). The observed difference in exclusions of prior CRCs (0.09%) is almost half as large as the reported difference in CRC risk (0.22%), which implies that such disproportional exclusions may have led to underestimation of screening effects on CRC risk by close to one third [20]. Furthermore, because participants in whom a previous CRC diagnosis may have been missed (e.g. by less than perfect identification of such cases in record linkage with cancer registries) are at substantially increased risk of CRC death compared to people with no previous diagnosis, differential exclusion rates of participants with a previous CRC diagnosis may have had an even stronger impact on the effect estimates on CRC mortality.

#### Outcome ascertainment bias: delayed and differential case ascertainment

Another potential bias that is well-known to potentially seriously threaten the validity of both observational epidemiological studies as well as RCTs is imperfect measurement of the outcome, such as differential ascertainment of disease events between exposure groups. In the NordICC trial, CRC case ascertainment during follow-up was made by record linkage with cancer registries.

Delayed completeness of cancer registry data by delayed notification to and processing of data within population-based cancer registries is well known, even for the highest quality cancer registries. For example, in a recent analysis of data from European population-based cancer registries, the median time from incidence to cancer registration for CRC was estimated between 600 and 700 days [21], i.e., close to 2 years. As shown in Fig. 1, 622 new CRC cases were identified during 10-year follow-up in the control group, on average 62 cases per year. More than twice that number, i.e.,164 participants who were originally randomized to the usual-care group and whose CRC diagnosis had yet remained undisclosed by the cancer registries at the time of randomization, had later to be excluded from the analysis. These numbers suggest an average delay of cancer registration of more than 2 years in the populations from which the NordICC study particiants were drawn. Hence, it is very plausible to assume substantial underascertainment of CRC cases that occurred during the late years of follow-up.

Because the proportion of CRC cases who were diagnosed in later follow-up years was much larger in the control group than in the invited group (in which prevalent cases were detected at screening colonoscopy among screening attenders), this differential underascertainment of cases is expected to be larger in the control group, eventually leading to major underestimation of screening effects. In fact, the reported reduction of CRC incidence in the invited group started to emerge after six years of follow-up only, i.e. it gradually evolved in the 4-year period from 6 to 10-year follow-up. As previously pointed out and illustrated in Table 4, underestimation of true screening effects by 2-year delay in cancer registration may therefore well have been in the order of 50% [22]. Hence, an updated analysis of 10-year results once 10-year follow-up can be considered reasonably complete is of paramount importance to overcome the expected bias due differential underascertainment of cases.

**Table 4:** Estimation of 10-year CRC risk reduction and numbers of people needed to invite for screening colonoscopy (intention-to-screen analysis) and numbers of people needed to undergo screening colonoscopy (adjusted per-protocol analysis) to prevent one CRC, assuming various degrees of mean delay in cancer registration in the NordICC trial

Analysis	Metric	Assumed mean delay in cancer registration			
		None^a^	1 year	2 years	3 years
Intention-to-screen	10-year risk difference	0.22% (0.07–0.37%)	0.29%	0.44%	0.88%
	Number needed to invite	455 (270–1429)	345	227	114
Adjusted per-protocol	10-year risk difference	0.38% (0.20–0.58%)	0.51%	0.76%	1.52%
	Number needed to scope	263 (172–500)	197	132	66

Results extracted from reference [22]

*CRC* colorectal cancer

^a^Results (95% CI) reported by Bretthauer et al. [1]

### Need for alternative analyses and additional information

#### Unravelling early-detection and prevention effects

**Fig. 2:**
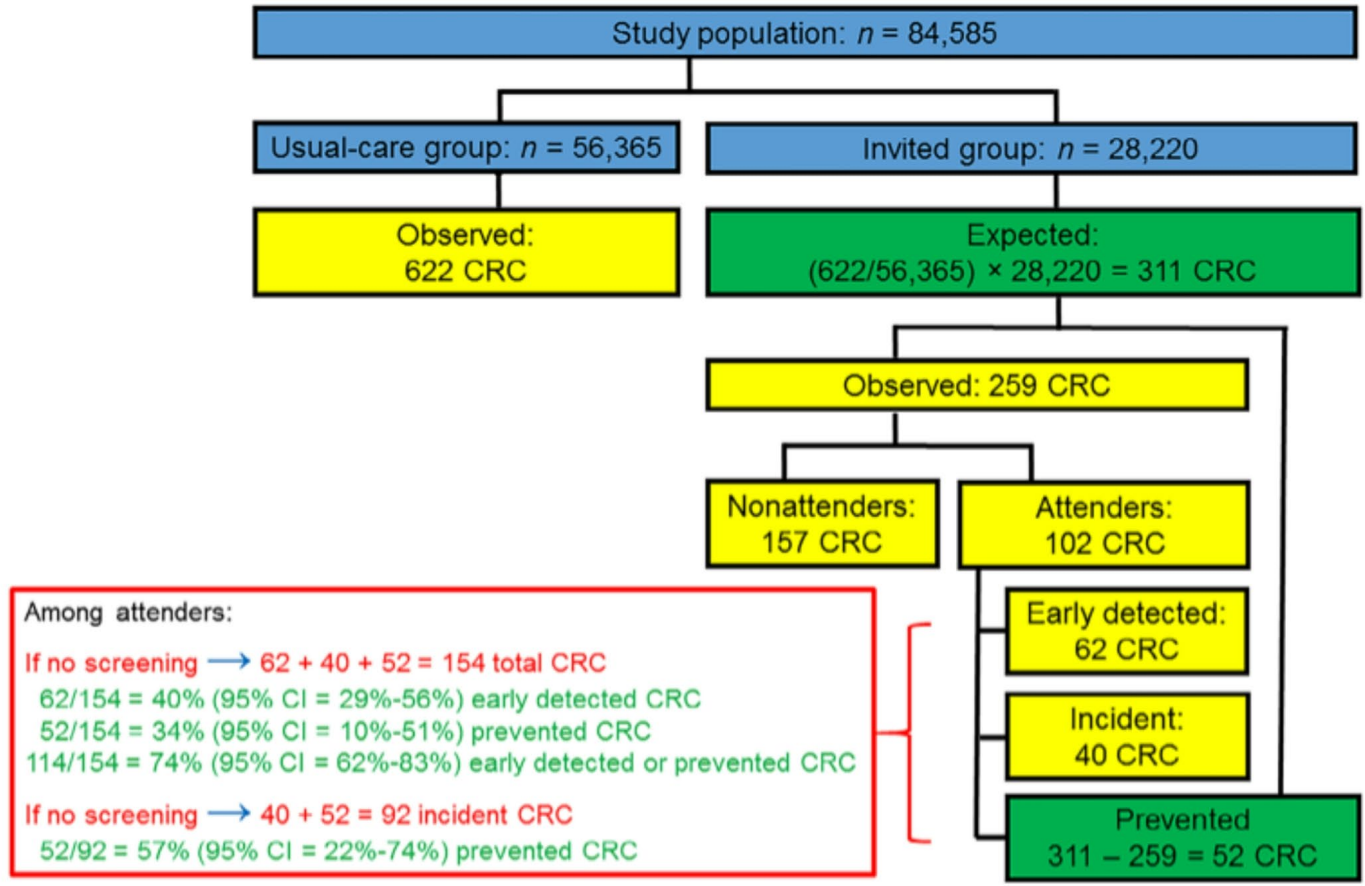
Derivation of the proportions of early-detected and prevented CRC cases among screening attenders from the NordICC trial (Figure originally published under the CC BY-NC-ND 4.0 licence as Fig. 1 in reference [26])

As previously pointed out [23, 24], results presented by the NordICC investigators do not differentiate screening effects on early detection of CRC cases that were already prevalent (but yet undiagnosed) at the time of recruitment and truly incident cases. Rather, both prevalent and incident cases were lumped together in reported "incidence" results [1]. In a previous commentary, Song and Bretthauer argued that "prevalent cancers at screening should be counted in clinical trials because there are no reliable statistical analyses which can tease out the true screening benefits without counting them" [25]. However, as recently demonstrated elsewhere [26] and illustrated in Fig. 2, it is possible to derive and unravel early detection and screening effects among screening attenders under two basic, plausible assumptios: (i) equal CRC risk in the intervention group and the usual care group (the “standard RCT assumption”), and (ii) screening colonoscopy can only prevent CRC among those who attended it (a "common sense" assumption). Under these assumptions, the proportions of CRC cases that were either early-detected (40%) or prevented (34%) among screening attenders were estimated as 74% from the published NordICC results, and the proportion of prevented incident cases was estimated as 57% [26]. We suggest this type of analysis as an important and more informative alternative to the various types of adjusted per-protocol analyses provided by Bretthauer and colleagues in their original NordICC trial report [1] and their follow-up article in this issue [2], whose results are summarized in Table 3.

It should be noticed, however, that all of the results shown in Fig. 2; Table 2 still underestimate true effects due to the various sources of bias outlined above and should be repeated after eliminating these biases by work-up and cleaning of the data to the best possible extent.

#### Disclosing the extent of contamination

Finally, it should be noted that the NordICC trial was conducted in a period in which colonoscopy outside screening offers became widely available and used. According to results from the European Health Interview Surveys conducted in 2013–2015 and 2019, between 20 and 40% of the population in the respective age group in the NordICC countries would be expected to have had a colonoscopy in the preceding 10 years [24]. Even the per-protocol estimates reported by Bretthauer et al. should therefore not be interpreted as comparing risks of CRC and CRC death of people with and without colonoscopy.

„Contamination“ of unscreened participants who had colonoscopies outside the trial during follow-up may strongly attenuate screening colonoscopy effect estimates [24, 27, 28]. Information on use of all types of colonoscopies (screening colonoscopies, surveillance colonoscopies, diagnostic colonoscopies) in both the invited and group and the control group, which should be readily available to the NordICC investigators, was not addressed in the NordICC trial report. Such information should be transparently and comprehensively included in future reports. This would allow more informed interpretation of the use and effects of various types of colonoscopy in the „colonoscopy era“.

## Conclusions

Avoidance of confounding is a unique asset of large-size RCTs compared to observational epidemiological studies, which has rendered the RCT design to be the holy grail for evaluating effectiveness of clinical intervention. However, RCTs may be prone to other sources of bias which can be as detrimental as confounding or in some circumstances even more detrimental and which require most careful attention in the study design and analyses and in the interpretation of results. The NordICC trial, the first and so far only RCT reporting on the effects of screening colonoscopy on CRC incidence and mortality, found much weaker (and in case of CRC mortality non-significant) effects than anticipated from a wealth of carefully conducted observational epidemiological studies that had quite consistently suggested strong protective effects. It has been very tempting to many to claim confounding and other biases in observational evidence as the main reason for this apparent discrepancy, and to conclude that screening colonoscopy was not as effective as presumed over the past 30 years.

A critical review of the NordICC trial design and results suggests, however, that other sources of bias, such as selection and information bias, which cannot be prevented by randomization and which can affect both observational studies and RCTs, may have led to major underestimation of screening colonoscopy effects in the NordICC trial and explain the lower-than-expected effects observed in the trial. One potential source of major bias, differential underascertainment of cases within 10 years of follow-up due to delayed ascertainment of CRC cases in record likage with cancer registries, may be remedied by repeating the analysis after having ensured sufficiently complete case ascertainment throughout the entire follow-up period. Furthermore, other potential sources of major bias, such as the significantly differential post-randomization exclusion rates, and ways to overcome the resulting bias should be carefully explored. Also, the extent of use of colonoscopies other than those specifically offered in the trial should be monitored and reported for both the invited group and the control group. Along with novel approaches of analysis that unravel and disentangle both the early detection and prevention effects of screening colonoscopy as well as more detailed country-specific analyses, this will enable a much more compelling contribution of this unique trial to valid judgment of the efficacy of screening colonoscopy in reducing the burden of CRC.

Implementation of screening colonoscopy following the landmark publication by Winawer et al. in 1993 [5] and subsequent observational epidemiological studies has prevented hundreds of thousands of CRC cases and deaths in some countries, such as the US and Germany [29–31]. Not engaging in any CRC sceening (for which there are effective alternative approaches besides screening colonoscopy, such as screening by fecal immunochemical tests [6, 32, 33]) has prompted a large toll of avoidable CRC cases and deaths in many other countries [7]. The momentum for CRC screening should by no means slowed by misinterpretation of the NordICC trial evidence.

## Abbreviations

CRC: Colorectal cancer
NordICC: Nordic-European Initiative on Colorectal Cancer
RCT: Randomized controlled trial
